# Gastrointestinal safety of etoricoxib in osteoarthritis and rheumatoid arthritis: A meta-analysis

**DOI:** 10.1371/journal.pone.0190798

**Published:** 2018-01-10

**Authors:** Xiaoting Feng, Mei Tian, Wei Zhang, Hong Mei

**Affiliations:** 1 Department of Nephrology and Rheumatology, Affiliated Hospital of Zunyi Medical College, Zunyi, Guizhou, China; 2 Department of Critical Care Medicine, Affiliated Hospital of Zunyi Medical College, Zunyi, Guizhou, China; University of Catanzaro, ITALY

## Abstract

**Objective:**

To ascertain if etoricoxib increases the risk of gastrointestinal adverse events (GAEs) compared with placebo, diclofenac, and naproxen in the treatment of patients with osteoarthritis (OA) or rheumatoid arthritis (RA).

**Methods:**

Studies were searched in MEDLINE, EMBASE, Cochrane Central Register of Controlled Trials from inception to August 2017. Randomized Clinical Trials (RCTs) that compared etoricoxib with placebo and other active drug for patients with OA or RA and reported data on gastrointestinal safety (which is of interest to patients and clinicians) were included. The follow-up time window for GAEs was defined as within 28 days subsequent to the last dose of study medication. A meta-analysis was conducted using a fixed-effect model. Risk ratios (RRs) and 95% confidence intervals (CIs) were measured.

**Results:**

We found nine randomized clinical trials (RCTs) that included information on gastrointestinal safety during follow-up time. Among them, five RCTs compared etoricoxib with placebo, four RCTs compared etoricoxib with diclofenac, and three RCTs compared etoricoxib with naproxen. Etoricoxib did not increase the risk of GAEs compared with placebo. Compared with diclofenac and naproxen, etoricoxib reduced the GAE risk (RR, 0.67; 95% CI, 0.59–0.76; p < 0.00001; 0.59; 0.48–0.72; < 0.00001) during follow-up time.

**Conclusions:**

In patients with OA or RA, etoricoxib did not increase the GAE risk compared with placebo, but reduced the GAE risk effectively compared with diclofenac and naproxen during follow-up time.

## Introduction

Osteoarthritis (OA) and rheumatoid arthritis (RA) are common diseases worldwide, as well as leading causes of morbidity and disability that threaten human health. In the USA, OA affected more than 27 million patients in 2008 (up from an estimate of 21 million in 1995, and 25 million in 1998).[1,2] The primary objectives of treatment for patients with OA or RA are to control pain, improve function, and reduce disability.[3] Of all the strategies available for the treatment of OA and RA, non-steroidal anti-inflammatory drugs (NSAIDs) are often prescribed to relieve pain and inflammation.[4–12] It has been estimated that 5% of all visits to a family doctor in the USA are related to prescriptions of NSAIDs, which are the most commonly used drugs in the USA.[13,14] Due to their inherent toxicity to the upper/lower gastrointestinal tract resulting from their additional inhibition of the cyclooxygenase (COX)-1, to some extent the use of traditional NSAIDs can be limited and recommended at the lowest possible dose and for short-term treatment.[15,16]

Selective COX-2 inhibitors, as a type of non-steroidal anti-inflammatory drug (NSAID), are able to directly target cyclooxygenase-2 (COX-2) an enzyme responsible for inflammation and pain leading to reduce the risk of peptic ulceration.

The inhibition of prostaglandin formation by COX inhibition is the major the mechanism of action of NSAIDs. COX exists in two major forms: COX-1 and COX-2. In patients with OA or RA, traditional NSAIDs such as diclofenac and naproxen relieve pain by inhibiting COX-1 and COX-2, but are also associated with a high prevalence of gastrointestinal adverse events (GAEs) resulting from inhibition of the gastro-protective COX-1. Therefore, an increasing demand for more effective and safer treatments for OA and RA has led to the development of newer selective COX-2 inhibitors such as etoricoxib. Such inhibitors have demonstrated efficacy at recommended doses for relieving pain and improving physical function in patients with OA or RA.[4–12]

After several COX-2 inhibiting drugs were approved by the US Food and Drug Administration, data from clinical trials revealed that COX-2 inhibitors led to a significant increase in heart attacks and strokes. Compared with other COX inhibitors, etoricoxib is an iterative drug used for relief of the pain associated with OA and RA. Randomized controlled trials (RCTs) focusing on the safety of etoricoxib have shown it to have suboptimal safety with regard to gastrointestinal and cardiovascular systems. Rofecoxib (commonly known as Vioxx) is marketed as a selective inhibitor of COX-2. In 2004, rofecoxib was withdrawn from the market because a randomized placebo-controlled trial showed an increased risk of cardiovascular events associated with this drug, even celecoxib and traditional NSAIDs received boxed warnings on their labels.[17] Also, the US Food and Drug Administration decided against the approval of etoricoxib due to its inadequate risk–benefit profile.[18] Many COX-2-specific inhibitors have been removed from the U.S. market. As of December 2011, only celecoxib is still available for purchase in the United States.

We undertook a study to ascertain if etoricoxib increases the risk of GAEs compared with placebo, diclofenac, and naproxen in the treatment of patients with OA or RA during follow-up time.

## Methods

### Search methods

Relevant studies were searched in MEDLINE, EMBASE, Cochrane Central Register of Controlled Trials from inception to August, 2017. We used the MESH terms “osteoarthritis, rheumatoid arthritis” and “cyclooxygenase-2 selective inhibitor, etoricoxib and Arcoxia” in the above database. We did not include additional unpublished data or unpublished studies. Studies were identified by the three authors of the present study, and relevant studies were selected by consensus.

### Selection criteria

We included all RCTs comparing placebo, diclofenac, and naproxen with etoricoxib for OA and RA. We assessed the risk of bias of the studies according to advice from the Cochrane Collaboration Review Group. This advice relied on the use of randomization and blinding to the intervention, and was also based on the completeness and blinding of follow-up.

For inclusion, the study had to: (1) include OA or RA, with the knee and/or hip being the affected joint; (2) compare etoricoxib with placebo, diclofenac, and naproxen in the treatment of OA or RA for ≥4 weeks; (3) provide endpoints for the gastrointestinal tolerability of etoricoxib, placebo, diclofenac and naproxen. Also, with regard to GAEs: (i) patients had to have suffered one or more upper-gastrointestinal event;[8] (ii) treatment had to be discontinued due to GAEs;[7,9,11,12] (iii) gastrointestinal “nuisance symptoms” had to be recorded.[4–6,10]

The outcome measure for gastrointestinal tolerability was GAEs in patients. Two independent reviewers extracted data from original studies.

An appropriate methodology of RCT design is defined as the whole methodological design of RCTs including the follows: 1) random sequence generation (selection bias); 2) allocation concealment (selection bias); 3) blinding participants and personnel (performance bias); 4) blinding of outcome assessment (detection bias); 5) incomplete outcome data (attrition bias); 6) selective reporting (reporting bias); 7) other bias.

### Data collection

In our meta-analysis, GAEs were the primary outcome in patients with OA or RA. The follow-up period for GAEs was within 28 days since the final dose of the study medication. We selected reports that included data evaluating the effect of the intervention on GAE prevalence.

### Data analyses

We extracted the data of AGEs including all the contents of the included criteria (3) and performed meta-analysis in the software of Review Manager v5.3.

### Statistical analyses

We undertook the meta-analysis using Review Manager v5.3 (Nordic Cochrane Centre, Copenhagen, Denmark) using the Mantel–Haenszel method and a fixed-effect model. We calculated risk ratios (RRs) for dichotomous variables, with 95% confidence intervals (CIs). All statistical tests were two-sided and analyses were not corrected for multiple comparisons. p < 0.05 was considered significant. We examined heterogeneity among studies using *χ*^2^ and *I*^2^ tests.

## Results

### Sample characteristics

**Fig 1:**
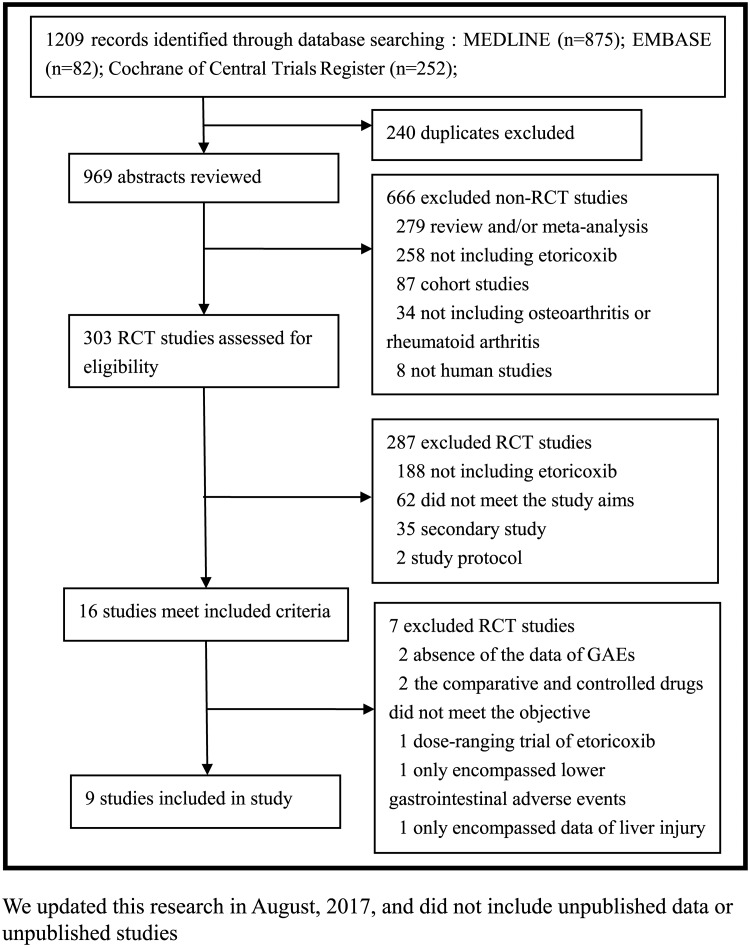
Flow-chart of study selection process.

Our literature search identified 1209 unique records (Fig 1). After omission of duplicated records, 969 records remained. Among them, 303 RCTs were assessed for eligibility, 287 RCTs were excluded for not including etoricoxib, not meeting the study aim, being a secondary study, or not stating the study protocol, and 16 RCTs included etoricoxib.[4–12] However, 7 RCTs were excluded due to absence of the data of GAEs, the comparative and controlled drugs did not meet the objective of the study, dose-ranging trial of etoricoxib, only encompassed lower gastrointestinal adverse events, or only encompassed data of liver injury.[19–25] Finally, 9 RCTs were included in the study. Among these nine RCTs, five encompassed GAEs in patients with OA or RA comparing etoricoxib with placebo[4,5,7,9,10], four RCTs compared etoricoxib with diclofenac[6,8,11,12], and three RCTs compared etoricoxib with naproxen[4,5,10].

We compiled a dataset of 2694 adult patients with OA or RA from five RCTs comparing etoricoxib with placebo[4,5,7,9,10], 21476 patients from four RCTs comparing etoricoxib with diclofenac[6,8,11,12], and 1864 patients from three RCTs comparing etoricoxib with naproxen[4,5,10], which at least included GAE data.

**Fig 2:**
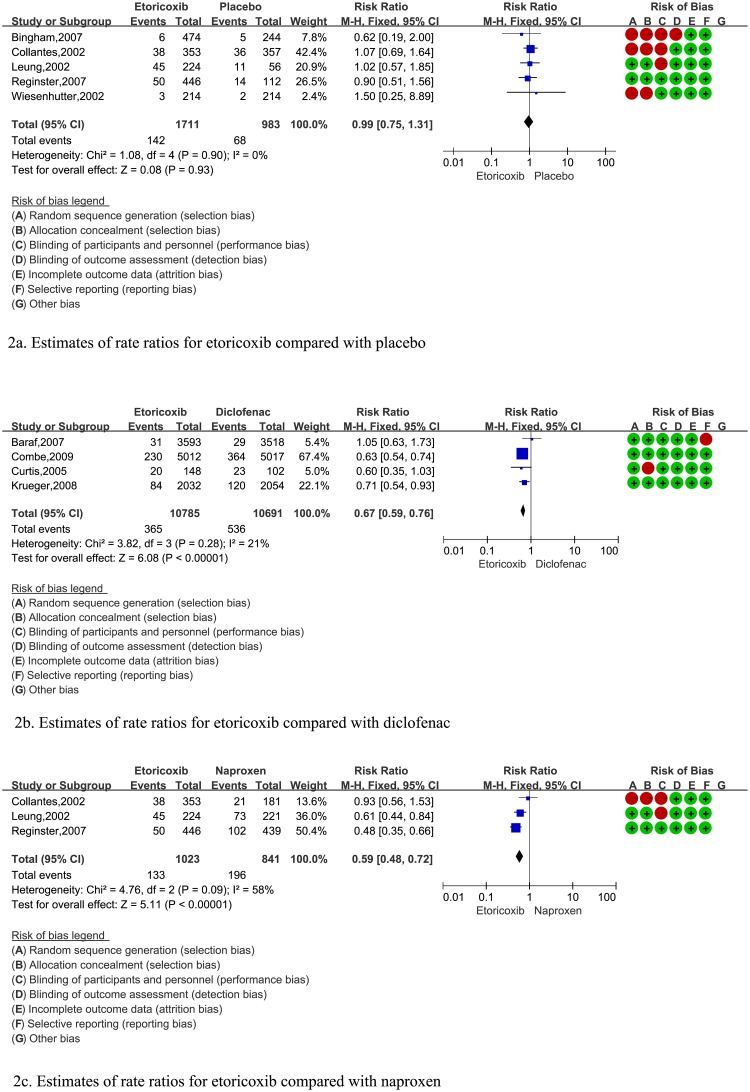
Forest plot of estimates of rate ratios for etoricoxib compared with placebo, diclofenac, and naproxen.

A summary of RCT characteristics is shown in Table 1. Of the nine RCTs included, six RCTs encompassed a population with OA, two RCTs focused in an RA population, and one RCT looked at OA patients and RA patients. Of the nine RCTs that reported the prevalence of GAEs during medication administration, three RCTs had caregivers who were not blinded to the treatment assignment, and four RCTs had an interventional population who did not have concealed allocation of the medication (Fig 2).

**Table 1:** Characteristics of included trials.

Trial	Interventions	Population	No of patients*	Follow-up (weeks)	Low dose aspirin allowed	Concealed allocation†	Blinding‡	Events adjudicated§	Intention to treat
Baraf,2007 (EDGE)	etoricoxib (90 mg/d) v Diclofenac (150 mg/d)	Osteoarthritis	7111	4	Yes	Yes	Yes	Yes	No
Bingham,2007	etoricoxib (30 mg/d)v celecoxib (200 mg/d) v Placebo	Osteoarthritis	1207	26	Yes	No	No	Yes	Yes
Collants,2002	etoricoxib (90 mg/d) v naproxen (1000 mg/d) v Placebo	rheumatoid arthritis	891	12	Yes	No	No	Yes	Yes
Combe,2009	etoricoxib (60 or 90 mg/d) v Diclofenac (150 mg/d)	rheumatoid arthritis and Osteoarthritis	23504	52	Yes	Yes	Yes	Yes	Yes
Curtis,2005	etoricoxib (30 or 60 or 90 mg/d) v Diclofenac (150 mg/d)	Osteoarthritis	617	52	Yes	No	Yes	Yes	Yes
Krueger,2008 (EDGE-II)	etoricoxib (90 mg/d) v Diclofenac (150 mg/d)	rheumatoid arthritis	4086	150	Yes	Yes	Yes	Yes	No
Leung,2002	etoricoxib (60 mg/d) v naproxen (1000 mg/d) v Placebo	Osteoarthritis	501	12	Yes	Yes	No	Yes	Yes
Reginster,2007	etoricoxib (60 mg/d) v naproxen (1000 mg/d)	Osteoarthritis	997	12	Yes	Yes	Yes	Yes	Yes
Wiesenhutter,2005	etoricoxib (30 mg/d) v ibuprofen (2400 mg/d) v Placebo	Osteoarthritis	528	12	Yes	No	Yes	Yes	Yes

* Number of randomized patients of included trial arms.

^†^Yes if investigators responsible for patient selection were unable to suspect before allocation which treatment was next in line (central randomization, sequentially numbered, sealed, opaque assignment envelopes, coded drug packs).

^‡^Yes if drugs looked similar (for example, matching placebo) or double dummy was used.

^§^Relates to GAEs only.

**Fig 3:**
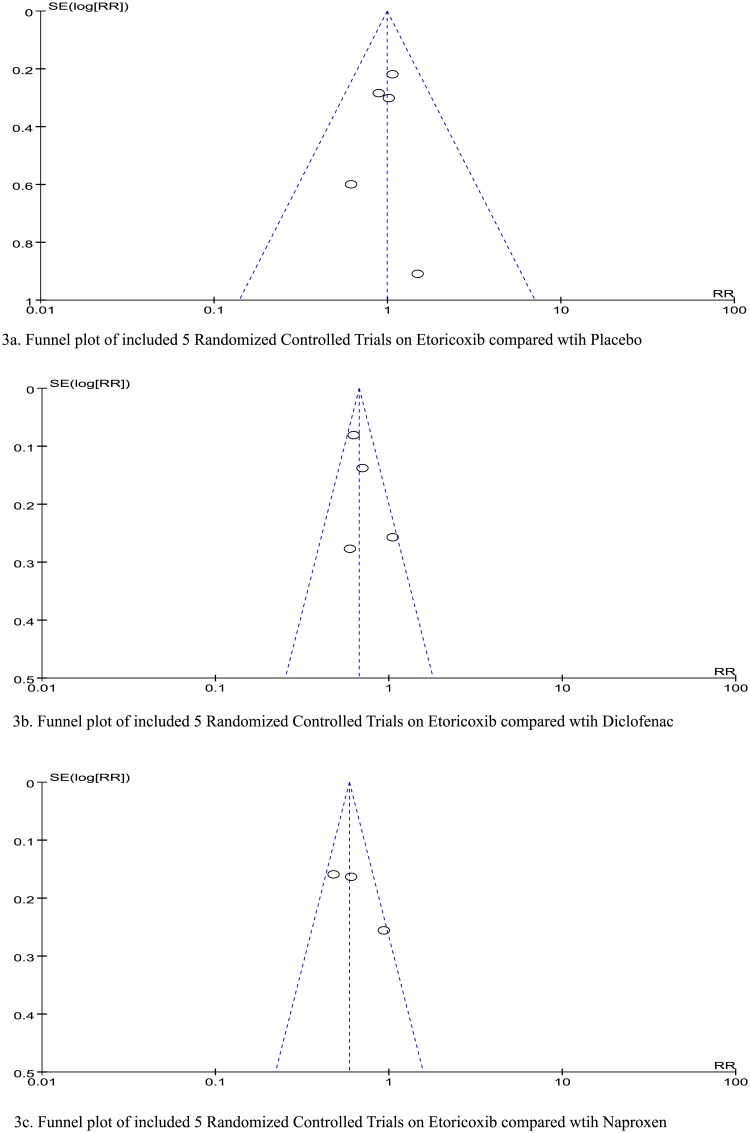
Funnel plot of publication bias for etoricoxib compared with placebo, diclofenac, and naproxen.

Funnel plots for publication bias with regard to GAEs during medication administration are shown for etoricoxib compared with placebo (Fig 3a), etoricoxib compared with diclofenac (Fig 3b) and etoricoxib compared with naproxen (Fig 3c). With regard to the methodologic quality of the RCTs included for GAE assessment, three RCTs used an appropriate methodology.

The results of data extraction is shown in Table 2. We identified five RCTs involving 2694 patients with OA or RA. These RCTs contained information about GAEs comparing etoricoxib with placebo during medication administration. Compared with placebo, etoricoxib did not increase the prevalence of GAEs (RR, 0.99; 95% CI, 0.75–1.31; p = 0.93) during medication administration (Fig 2a). We assessed the risk of bias on the 5 RCTs of GAEs of etoricoxib compared with placebo. 3 RCTs including Bingham 2007, Collantes 2002, and Wiesenhutter 2002 did not encompass the methods of the follows: random sequence generation (selection bias) and allocation concealment (selection bias); 3 RCTs including Bingham 2007, Collantes 2002, and Leung 2002 did not encompass the methods of blinding of participants and personnel (performance bias); and one RCT did not encompass the methods of blinding of outcome assessment (detection bias). Finally, one RCT performed the appropriate methods of randomized controlled trials.

**Table 2:** Incidence of confirmed adverse events of gastrointestinal (GI) safety.

Trial	Etoricoxib		Placebo		Diclofenac		Naproxen	
	Events	No of patients	Events	No of patients	Events	No of patients	Events	No of patients
Baraf,2007† (EDGE)	31	3593			29	3518		
Bingham,2007*	6	474	5	244				
Collants,2002*‡	38	353	36	357			21	181
Combe,2009†	230	5012			364	5017		
Curtis,2005†	20	148			23	102		
Krueger,2008† (EDGE-II)	84	2032			120	2054		
Leung,2002*‡	45	224	11	56			73	221
Reginster,2007*‡	50	446	14	112			102	439
Wiesenhutter,2005*	3	214	2	104				
Total	507	12496	68	873	536	10691	196	841

* Etoricoxib compared with placebo

^†^ Etoricoxib compared with diclofenac

^‡^ Etoricoxib compared with naproxen

Among the four RCTs involving 21476 patients with OA or RA comparing etoricoxib with diclofenac, etoricoxib reduced the GAE risk significantly (RR, 0.67; 95% CI, 0.59–0.76; p < 0.00001). Moreover, among the three RCTs involving 1864 patients with OA or RA comparing etoricoxib with naproxen, etoricoxib reduced the GAE risk significantly (RR, 0.59; 95% CI, 0.48–0.72; p < 0.00001).

We also assessed the risk bias of the 4 RCTs for etoricoxib compared with diclofenac, and we found that 2 RCTs performed the appropriate methods but Curtis 2005 that did not encompass the methods of allocation concealment. Finally, Among the risk bias of 3 RCTs compared etoricoxib with naproxen, we found that only Reginster 2007 performed the appropriate methods of RCTs, while the left two RCTs including Leung 2002 and Collantes 2002 had been assessed in the above two studies on etoricoxib compared with placebo and diclofenac.

In the assessment of publication bias (Fig 3),we found that the 3 separated studies including etoricoxib compared with placebo (Fig 3a), diclofenac (Fig 3b), and naproxen (Fig 3c) did not occur in publication bias.

## Discussion

We undertook a meta-analysis of gastrointestinal-safety data for etoricoxib and placebo, diclofenac, and naproxen. We found that etoricoxib could reduce the risk of GAEs compared with diclofenac and naproxen, but was equivalent to placebo, for patients with OA or RA.

Several systematic reviews and meta-analyses of etoricoxib have been carried out,[3,26–31] but few studies have focused on the efficacy of etoricoxib in the treatment of OA and RA. In 2005, a systemic review and meta-analysis on the thromboembolic cardiovascular events associated with etoricoxib by Aldington and colleagues suggested that etoricoxib increased the risk of cardiovascular events (odds ratio, 1.49; 95% CI, 0.42–5.31),[26] but GAEs were not evaluated. In 2016, a Bayesian network meta-analysis of RCTs by Song and co-workers suggested no significant difference in tolerability between etoricoxib and naproxen.[3] The negative results obtained by Aldington and colleagues and Song and co-workers differed from our data due to three main reasons. First, their studies on the tolerability of etoricoxib compared with naproxen encompassed only OA patients, whereas we also included RA patients. Second, in their studies, the dose of etoricoxib was 60 mg, whereas it was 30mg, 60mg, and 90 mg in our study. Finally, in their studies, the tolerability of drugs encompassed adverse cardiovascular events and GAEs, whereas we focused only on GAEs.

### Gastrointestinal safety of etoricoxib compared with placebo

The result of that meta-analysis produce point estimates was negative in the five randomized controlled trials on gastrointestinal safety of etoricoxib compared with placebo in the treatment of OA or RA. However, the conclusion of each study seemed completely inconsistent. For example, Wiesenhutter and colleagues[7] concluded that etoricoxib increased the risk of GAEs compared with placebo, whereas Bingham and colleagues[9] drew the conclusion of decreasing the risk of GAEs. Simultaneously, the statistical power of two studies was poor due to small sample sizes. The results of studies by Collantes and colleagues[4], Leung and coworkers[5], and Reginster and colleagues[10] all suggested that etoricoxib did not increase the risk of GAEs compared with placebo. The results of a meta-analysis of these five RCTs concluded that etoricoxib did not increase the risk of GAEs compared with placebo, and produced a point estimate of 0.99 (95% CI, 0.75–1.31).

### Gastrointestinal safety of etoricoxib compared with that of diclofenac

Four RCTs focused on the gastrointestinal safety of etoricoxib compared with that of diclofenac. The results of the RCTs conducted by Curtis and colleagues[6], Krueger and coworkers[11], and Combe and colleagues[12] suggested that etoricoxib reduced the GAE risk compared with diclofenac, but the RCT by Baraf and coworkers[8] did not arrive at this conclusion. A meta-analysis of these four RCTs produced a point estimate of 0.67 (95% CI, 0.59–0.76).

### Gastrointestinal safety of etoricoxib compared with that of naproxen

Three RCTs looked at the gastrointestinal safety of etoricoxib compared with that of naproxen. The results of the RCTs conducted by Leung and colleagues[5] and Reginster and coworkers[10] suggested that etoricoxib reduced the risk of GAEs compared with that by diclofenac, but Collantes and colleagues[4] did not arrive at this conclusion. A meta-analysis of these three RCTs produced a point estimate of 0.59 (95% CI, 0.48–0.72).

### Strengths of our meta-analysis

Our meta-analysis had three main strengths. First, only a single variable (GAE) was selected and used for the meta-analysis and, to a certain extent, it precluded confounding factors from other variables. Second, the variable of GAE is easy to measure during medication administration in patients with OA or RA, which reduces the risk of dropout from a RCT. Finally, we undertook a meta-analysis of GAEs comparing not only etoricoxib with placebo, but also diclofenac and naproxen.

### Weaknesses of our meta-analysis

Our meta-analysis had six main limitations. First, the sample size of GAEs in RCTs comparing etoricoxib with placebo and etoricoxib with naproxen was relatively small, so the statistical power of the results was limited. Second, the different therapeutic doses and follow-up durations of etoricoxib in these nine RCTs would have affected the results. Third, the quality of the RCTs (including random-sequence generation, allocation concealment, blinding of participants/personnel/outcome assessment) were suboptimal. Fourth, most of the RCTs in our meta-analysis were not recent. Fifth, GAEs are usually regarded a secondary outcome in studies, so there are few studies related to this topic. Finally, different RCTs had different definitions of GAE, which would affect the conclusions reached. We defined GAEs (excluding laboratory-based GAEs) as clinical events encompassing gastrointestinal nuisance symptoms that led to treatment discontinuation.

### Generalizability of our results

Although our meta-analysis had the limitations mentioned above, we can conclude that etoricoxib is relatively safe for administration in patients with OA or RA, and can be used widely in these populations.

## Conclusions

In patients with OA or RA, etoricoxib did not increase the GAE risk compared with placebo, but reduced the GAE risk effectively compared with diclofenac and naproxen during follow-up time.

## Supporting information

S1 Fig PRISMA 2009 flow diagram. (DOC)

S1 Table PRISMA 2009 checklist. (DOC)

## References

[1] Lawrence RC, Felson DT, Helmick CG, Arnold LM, Choi H, Deyo RA, et al (2008) Estimates of the prevalence of arthritis and other rheumatic conditions in the United States. Part II. Arthritis Rheum 58: 26–35. doi: 10.1002/art.23176 18163497 10.1002/art.23176 PMC3266664

[2] Lawrence RC, Helmick CG, Arnett FC, Deyo RA, Felson DT, Giannini EH, et al (1998) Estimates of the prevalence of arthritis and selected musculoskeletal disorders in the United States. Arthritis Rheum 41: 778–799. doi: 10.1002/1529-0131(199805)41:5<778::AID-ART4>3.0.CO;2-V 9588729 10.1002/1529-0131(199805)41:5<778::AID-ART4>3.0.CO;2-V

[3] Song GG, Seo YH, Kim JH, Choi SJ, Ji JD, Lee YH, et al (2016) Relative efficacy and tolerability of etoricoxib, celecoxib, and naproxen in the treatment of osteoarthritis: A Bayesian network meta-analysis of randomized controlled trials based on patient withdrawal. Z Rheumatol 75: 508–516. doi: 10.1007/s00393-015-0023-9 26768273 10.1007/s00393-015-0023-9

[4] Collantes E, Curtis SP, Lee KW, Casas N, McCarthy T, Melian A, et al (2002) A multinational randomized, controlled, clinical trial of etoricoxib in the treatment of rheumatoid arthritis [ISRCTN25142273]. BMC Fam Pract 3: 10 doi: 10.1186/1471-2296-3-10 12033987 10.1186/1471-2296-3-10 PMC115849

[5] Leung AT, Malmstrom K, Gallacher AE, Sarembock B, Poor G, Beaulieu A, et al (2002) Efficacy and tolerability profile of etoricoxib in patients with osteoarthritis: A randomized, double-blind, placebo and active-comparator controlled 12-week efficacy trial. Curr Med Res Opin 18: 49–58. doi: 10.1185/030079902125000282 12017209 10.1185/030079902125000282

[6] Curtis SP, Bockow B, Fisher C, Olaleye J, Compton A, Ko AT, et al (2005) Etoricoxib in the treatment of osteoarthritis over 52-weeks: a double-blind, active-comparator controlled trial [NCT00242489]. BMC Musculoskelet Disord 6: 58 doi: 10.1186/1471-2474-6-58 16321158 10.1186/1471-2474-6-58 PMC1327669

[7] Wiesenhutter CW, Boice JA, Ko A, Sheldon EA, Murphy FT, Wittmer BA, et al (2005) Evaluation of the comparative efficacy of etoricoxib and ibuprofen for treatment of patients with osteoarthritis: A randomized, double-blind, placebo-controlled trial. Mayo Clin Proc 80: 470–479. 15819283 10.4065/80.4.470

[8] Baraf HS, Fuentealba C, Greenwald M, Brzezicki J, O'Brien K, Soffer B, et al (2007) Gastrointestinal side effects of etoricoxib in patients with osteoarthritis: results of the Etoricoxib versus Diclofenac Sodium Gastrointestinal Tolerability and Effectiveness (EDGE) trial. J Rheumatol 34: 408–420. 17304660

[9] Bingham CO 3rd, Sebba AI, Rubin BR, Ruoff GE, Kremer J, Bird S, et al (2007) Efficacy and safety of etoricoxib 30 mg and celecoxib 200 mg in the treatment of osteoarthritis in two identically designed, randomized, placebo-controlled, non-inferiority studies. Rheumatology (Oxford) 46: 496–507. 16936327 10.1093/rheumatology/kel296

[10] Reginster JY, Malmstrom K, Mehta A, Bergman G, Ko AT, Curtis SP, et al (2007) Evaluation of the efficacy and safety of etoricoxib compared with naproxen in two, 138-week randomised studies of patients with osteoarthritis. Ann Rheum Dis 66: 945–951. doi: 10.1136/ard.2006.059162 17142385 10.1136/ard.2006.059162 PMC1955093

[11] Krueger K, Lino L, Dore R, Radominski S, Zhang Y, Kaur A, et al (2008) Gastrointestinal tolerability of etoricoxib in rheumatoid arthritis patients: results of the etoricoxib vs diclofenac sodium gastrointestinal tolerability and effectiveness trial (EDGE-II). Ann Rheum Dis 67: 315–322. doi: 10.1136/ard.2007.082388 17965424 10.1136/ard.2007.082388

[12] Combe B, Swergold G, McLay J, McCarthy T, Zerbini C, Emery P, et al (2009) Cardiovascular safety and gastrointestinal tolerability of etoricoxib vs diclofenac in a randomized controlled clinical trial (The MEDAL study). Rheumatology (Oxford) 48: 425–432. 19223284 10.1093/rheumatology/kep005

[13] Dai C, Stafford RS, Alexander GC. (2005) National trends in cyclooxygenase-2 inhibitor use since market release: nonselective diffusion of a selectively cost-effective innovation. Arch Intern Med 165: 171–177. doi: 10.1001/archinte.165.2.171 15668363 10.1001/archinte.165.2.171

[14] Kaufman DW, Kelly JP, Rosenberg L, Anderson TE, Mitchell AA. (2002) Recent patterns of medication use in the ambulatory adult population of the United States: the Slone survey. JAMA 287: 337–344. 11790213 10.1001/jama.287.3.337

[15] Laine L, Connors LG, Reicin A, Hawkey CJ, Burgos-Vargas R, Schnitzer TJ, et al (2003) Serious lower gastrointestinal clinical events with nonselective NSAID or coxib use. Gastroenterology 124: 288–292. doi: 10.1053/gast.2003.50054 12557133 10.1053/gast.2003.50054

[16] Singh G, Triadafilopoulos G. (1999) Epidemiology of NSAID induced gastrointestinal complications. J Rheumatol Suppl 56: 18–24. 10225536

[17] Bresalier RS, Sandler RS, Quan H, Bolognese JA, Oxenius B, Horgan K, et al (2005) Cardiovascular events associated with rofecoxib in a colorectal adenoma chemoprevention trial. N Engl J Med 352: 1092–1102. doi: 10.1056/NEJMoa050493 15713943 10.1056/NEJMoa050493

[18] Avorn J. (2007) Keeping science on top in drug evaluation. N Engl J Med 357: 633–635. doi: 10.1056/NEJMp078134 17699813 10.1056/NEJMp078134

[19] Gottesdiener K, Schnitzer T, Fisher C, Bockow B, Markenson J, Ko A, et al (2002) Results of a randomized, dose-ranging trial of etoricoxib in patients with osteoarthritis. Rheumatology (Oxford) 41: 1052–1061. 12209041 10.1093/rheumatology/41.9.1052

[20] Hunt RH, Harper S, Watson DJ, Yu C, Quan H, Lee M, et al (2003) The gastrointestinal safety of the COX-2 selective inhibitor etoricoxib assessed by both endoscopy and analysis of upper gastrointestinal events. Am J Gastroenterol 98: 1725–1733. doi: 10.1111/j.1572-0241.2003.07598.x 12907325 10.1111/j.1572-0241.2003.07598.x

[21] Laine L, Curtis SP, Langman M, Jensen DM, Cryer B, Kaur A, et al (2008) Lower gastrointestinal events in a double-blind trial of the cyclo-oxygenase-2 selective inhibitor etoricoxib and the traditional nonsteroidal anti-inflammatory drug diclofenac. Gastroenterology 135: 1517–1525. doi: 10.1053/j.gastro.2008.07.067 18823986 10.1053/j.gastro.2008.07.067

[22] Laine L, Goldkind L, Curtis SP, Connors LG, Yanqiong Z, Cannon CP, et al (2009) How common is diclofenac-associated liver injury? Analysis of 17,289 arthritis patients in a long-term prospective clinical trial. Am J Gastroenterol 104: 356–362. doi: 10.1038/ajg.2008.149 19174782 10.1038/ajg.2008.149

[23] Matsumoto AK, Melian A, Mandel DR, McIlwain HH, Borenstein D, Zhao PL, et al (2002) A randomized, controlled, clinical trial of etoricoxib in the treatment of rheumatoid arthritis. J Rheumatol 29: 1623–1630. 12180720

[24] Puopolo A, Boice JA, Fidelholtz JL, Littlejohn TW, Miranda P, Berrocal A, et al (2007) A randomized placebo-controlled trial comparing the efficacy of etoricoxib 30 mg and ibuprofen 2400 mg for the treatment of patients with osteoarthritis. Osteoarthritis Cartilage 15: 1348–1356. doi: 10.1016/j.joca.2007.05.022 17631392 10.1016/j.joca.2007.05.022

[25] Yoo MC, Yoo WH, Kang SB, Park YW, Kim SS, Moon KH, et al (2014) Etoricoxib in the treatment of Korean patients with osteoarthritis in a double-blind, randomized controlled trial. Curr Med Res Opin 30: 2399–2408. doi: 10.1185/03007995.2014.955169 25133963 10.1185/03007995.2014.955169

[26] Aldington S, Shirtcliffe P, Weatherall M, Beasley R. (2005) Systematic review and meta-analysis of the risk of major cardiovascular events with etoricoxib therapy. N Z Med J 118: U1684 16224508

[27] De Vecchis R, Baldi C, Di Biase G, Ariano C, Cioppa C, Giasi A, et al (2014) Cardiovascular risk associated with celecoxib or etoricoxib: a meta-analysis of randomized controlled trials which adopted comparison with placebo or naproxen. Minerva Cardioangiol 62: 437–448. 25029569

[28] Moore RA, Derry S, McQuay HJ. (2008) Discontinuation rates in clinical trials in musculoskeletal pain: meta-analysis from etoricoxib clinical trial reports. Arthritis Res Ther 10: R53 doi: 10.1186/ar2422 18466615 10.1186/ar2422 PMC2483442

[29] Moore RA, Moore OA, Derry S, Peloso PM, Gammaitoni AR, Wang H, et al (2010) Responder analysis for pain relief and numbers needed to treat in a meta-analysis of etoricoxib osteoarthritis trials: bridging a gap between clinical trials and clinical practice. Ann Rheum Dis 69: 374–379. doi: 10.1136/ard.2009.107805 19364730 10.1136/ard.2009.107805 PMC2800200

[30] Moore RA, Straube S, Paine J, Derry S, McQuay HJ. (2011) Minimum efficacy criteria for comparisons between treatments using individual patient meta-analysis of acute pain trials: examples of etoricoxib, paracetamol, ibuprofen, and ibuprofen/paracetamol combinations after third molar extraction. Pain 152: 982–989. doi: 10.1016/j.pain.2010.11.030 21414722 10.1016/j.pain.2010.11.030

[31] Zhang S, Zhang Y, Liu P, Zhang W, Ma JL, Wang J, et al (2016) Efficacy and safety of etoricoxib compared with NSAIDs in acute gout: a systematic review and a meta-analysis. Clin Rheumatol 35: 151–158. doi: 10.1007/s10067-015-2991-1 26099603 10.1007/s10067-015-2991-1

