# Supplementary material for: Gastrointestinal safety of etoricoxib in osteoarthritis and rheumatoid arthritis: A meta-analysis
Source: PLoS One. 2018 Jan 10;13(1):e0190798. doi: 10.1371/journal.pone.0190798 (PMC5761870; doi:10.1371/journal.pone.0190798)
Supplement: S1 Fig — (DOC) [file pone.0190798.s001.doc]

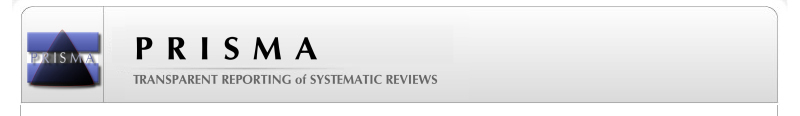
**PRISMA 2009 Flow Diagram**

**Screening**

**Included**

**Eligibility**

**Identification**

Records identified through database searching
(n =1209 )

Additional records identified through other sources
(n = 0 )

Records after duplicates removed
(n = 1209 )

Records screened
(n = 969 )

Records excluded
(n = 666 )

Full-text articles assessed for eligibility
(n = 303 )

Full-text articles excluded, with reasons
(n = 287 )

Studies included in qualitative synthesis
(n = 16 )

Studies included in quantitative synthesis (meta-analysis)
(n =9 )
